# MASCC/ISOO expert opinion on the management of oral problems in patients with advanced cancer

**DOI:** 10.1007/s00520-022-07211-2

**Published:** 2022-06-18

**Authors:** Jac A. Jones, Yanin Chavarri-Guerra, Luisa Barreto Costa Corrêa, David R. Dean, Joel B. Epstein, Eduardo R. Fregnani, Jiyeon Lee, Yuhei Matsuda, Valeria Mercadante, Ragnhild Elisabeth Monsen, Natasja J. H. Rajimakers, Deborah Saunders, Enrique Soto-Perez-de-Celis, Mariana S. Sousa, Arghavan Tonkaboni, Arjan Vissink, Keng Soon Yeoh, Andrew N. Davies

**Affiliations:** 1 Royal Surrey County Hospital, Guildford, UK; 2 Department of Hemato-Oncology, Instituto Nacional de Ciencias Médicas y Nutrición Salvador Zubirán, Mexico City, Mexico; 3 State Health Department of the Federal District, Brasilia, Brazil; 4 Department of Oral Medicine, University of Washington/Seattle Cancer Care Alliance, Seattle, USA; 5 City of Hope Comprehensive Cancer Center, Duarte & Cedars Sinai Health System, Los Angeles, USA; 6 Instituto Sírio-Libanês de Ensino E Pesquisa, Sao Paulo, Brazil; 7 College of Nursing & Mo-Im Kim Nursing Research Institute, Yonsei University, Seoul, South Korea; 8 Department of Oral and Maxillofacial Surgery, Shimane University Faculty of Medicine, Izumo, Japan; 9 Royal National ENT & Eastman Dental Hospitals, London, UK; 10 Department of Medicine, Lovisenberg Diaconal Hospital & Department for Interdisciplinary Health Sciences, University of Oslo, Oslo, Norway; 11 Netherlands Comprehensive Cancer Organisation (IKNL), Utrecht, The Netherlands; 12 Department of Dental Oncology, Health Services North, Northern Ontario School of Medicine, Sudbury, Canada; 13 Department of Geriatrics, Instituto Nacional de Ciencias Médicas y Nutrición Salvador Zubirán, Mexico City, Mexico; 14 IMPACCT, Faculty of Health, University of Technology Sydney, Sydney, Australia; 15 Department of Oral Medicine, School of Dentistry, Tehran University of Medical Sciences, Tehran, Iran; 16 Department of Oral and Maxillofacial Surgery, University Medical Center Groningen, University of Groningen, Groningen, Netherlands; 17 Special Needs Dental Unit, South Australia Dental Service, Adelaide, Australia; 18 Trinity College Dublin, University College Dublin & Our Lady’s Hospice Dublin, Trinity College Dublin, Dublin, Ireland

**Keywords:** Oral hygiene, Oral problems, Oral manifestations, Neoplasms, Palliative care, Practice guideline

## Abstract

**Purpose:**

The Palliative Care Study Group in conjunction with the Oral Care Study Group of the Multinational Association for Supportive Care in Cancer (MASCC) formed a sub-group to develop evidence-based guidance on the management of common oral problems in patients with advanced cancer.

**Methods:**

This guidance was developed in accordance with the MASCC Guidelines Policy. A search strategy for Medline was developed, and the Cochrane Database of Systematic Reviews and the Cochrane Central Register of Controlled Trials were explored for relevant reviews and trials, respectively. Guidance was categorised by the level of evidence, and “category of guideline” (i.e., “recommendation”, “suggestion” or “no guideline possible”).

**Results:**

Twelve generic suggestions (level of evidence – 5), three problem-specific recommendations and 14 problem-specific suggestions were generated. The generic suggestions relate to oral hygiene measures, assessment of problems, principles of management, re-assessment of problems and the role of dental/oral medicine professionals.

**Conclusions:**

This guidance provides a framework for the management of common oral problems in patients with advanced cancer, although every patient requires individualised management.

## Introduction

Oral symptoms/conditions (“oral problems”) are common in patients with advanced cancer and are the cause of significant morbidity in this group of patients [1]. Hence, it is essential that healthcare professionals involved in the care of patients with advanced cancer have a good understanding about the principles of oral hygiene/care and knowledge of the evidence-based management of common oral problems.

Observational studies suggest that oral problems are not well-managed in patients with advanced cancer [2]. The reasons for the latter are multiple and include inadequate assessment, lack of diagnosis, inappropriate treatment and inadequate re-assessment. Indeed, oral problems are often not assessed by healthcare professionals and importantly often not reported by patients and carers (and represent so-called orphan problems) [3]. Additionally, there are no up-to-date, evidence-based guidelines on the management of oral problems in patients with advanced cancer within the medical and dental literature.

On the basis of the above, the Palliative Care Study Group, in conjunction with the Oral Care Study Group, of the Multinational Association for Supportive Care in Cancer (MASCC) formed a sub-group (“Group”) to develop MASCC/ISOO (International Society of Oral Oncology) evidence-based guidance on the management of common oral problems in patients with advanced cancer. This paper provides an overview of oral problems in patients with advanced cancer, the methodology involved in developing this guidance and the evidence identified in support of the recommendations/suggestions within this guidance.

It should be noted that this guidance purposely does not cover specific cancer treatment–related oral problems, since MASCC/ISOO has already produced a series of evidence-based guidelines on these oral problems (e.g. oral mucositis, salivary gland hypofunction/xerostomia) [4–6].

## Background

### Aetiology

Oral problems may be related to either (a) a direct (“anatomical”) effect of the cancer, e.g. oral discomfort due to intraoral cancer; (b) an indirect (“physiological”) effect of the cancer—see below; (c) an effect of cancer treatment, e.g. salivary gland hypofunction due to head and neck radiotherapy; (d) an effect of a coexisting disease or its treatment or (e) a combination of the above factors [1].

Advanced cancer is associated with a number of sequelae that can indirectly result in oral problems, including physical problems such as fatigue (resulting in difficulty in undertaking oral hygiene), psychological problems such as depression (resulting in lack of motivation/difficulty in undertaking oral hygiene) [7] and relative immunosuppression (resulting in increased risk of oral infections) [8].

### Epidemiology

As discussed above, oral symptoms are common in patients with advanced cancer [9–13]. Table 1 shows the results for oral symptoms from a recent study of patients with advanced cancer receiving specialist palliative care in the UK [13]. In this study, 97.5% of patients had at least one oral symptom, and the mean number of oral symptoms was five (range 1–18). Table 2 shows the results for other oral conditions from relevant palliative care studies [9–12, 14–19]. Certain oral problems appear to become more common as the patient’s condition progresses (e.g. xerostomia, oral candidosis) [16, 20].

**Table 1:** Prevalence of oral symptoms in a cohort of patients with advanced cancer receiving specialist palliative care [13]

Oral symptom	Number of patients (*n* = 250)
“Dry mouth”	209 (83.5%)
“Taste disturbance”	139 (55.5%)
“Coating of tongue”	117 (47.0%)
“Lip discomfort”	96 (38.5%)
“Dirty mouth”	88 (35.0%)
“Difficulty swallowing”	86 (34.5%)
“Cracking of lips”	85 (34.0%)
“Mouth discomfort/pain”	76 (30.5%)
“Difficulty speaking”	68 (27.0%)
“Difficulty chewing”	57 (23.0%)
“Cracking of corner of mouth”	56 (22.5%)
“Sensitivity of teeth”	52 (21.0%)
“Ulcers in mouth”	42 (17.0%)
“Bad breath”	41 (16.5%)
“Jagged teeth”	41 (16.5%)
“Altered sensation in mouth”	27 (11.0%)
“Denture fitting problems”	26 (10.0%)
“Toothache/pain in teeth”	25 (10.0%)
“Burning sensation in mouth”	24 (9.5%)
“Bleeding from mouth”	19 (7.5%)

**Table 2:** Prevalence of oral conditions in patients with advanced cancer [9–12, 14–19]

Oral problem	Prevalence
Salivary gland hypofunction	82–83% [14, 15]
Oral candidosis	13–34%* [10, 11, 16, 17]
Dental caries	20–51% [9, 11, 18]
Gingivitis	11–100% [11, 18, 19]
Other oral infections	Relatively uncommon – no data
Aphthous stomatitis (“aphthous ulceration”)	14% [10]
Non-specific ulceration	12–26% [9, 12, 19]
Other dental problems	Relatively common – limited data [19]
Poor denture retention	57–83% [9, 18, 19]
Poor denture hygiene	27–55% [9, 19]

^*^Studies involving microbiology investigations

### Clinical features

The mouth is integral to various everyday functions, such as respiration, communication (i.e. verbal, non-verbal), eating and drinking, administration of medication (i.e. oral route, oral transmucosal route) and prevention of infection. Oral problems are a significant direct cause of morbidity in many patients receiving palliative care [13]. Moreover, oral problems can lead to a more generalised deterioration in a patient’s condition [21] and health-related quality of life [12]. Oral problems are also an indirect cause of mortality in some patients receiving palliative care (e.g. oral colonisation/infection leading to respiratory and systemic infections) [8, 22].

Oral problems often co-exist [13], and a sentinel problem can lead to the development of a number of other local and systemic problems (which magnifies the impact of the problem on the patient’s health-related quality of life). For example, salivary gland hypofunction (dry mouth) is associated with oral discomfort, lip discomfort, cracking of lips, taste disturbance (dysgeusia), difficulty chewing (dysmasesia), difficulty swallowing (dysphagia), decreased intake of nutrition, oesophagitis, difficulty speaking (dysphonia), anorexia, poor oral hygiene, halitosis, oral candidosis, dental caries, salivary gland infections (sialadenitis), dental demineralisation (causing dental sensitivity), denture fitting problems, sleep disturbance, embarrassment, anxiety, depression and social isolation [1, 4]. Importantly, the successful management of the sentinel problem may lead to the resolution of some/all of the other local and systemic problems.

Oral problems also impact relationships with family members and others, with some patients avoiding situations that may highlight the problem (e.g. eating with others), and other patients avoiding social interactions altogether [21, 23].

## Methods

The aim of the Group was to develop clinically relevant, evidence-based guidance on the management of common oral problems in patients with advanced cancer. Thus, it was agreed that the recommendations could include ones supported by “high” levels of evidence (e.g. systematic reviews), as well as ones supported by “low” levels of evidence (e.g. expert opinion), if the topic was deemed to be clinically relevant and stronger evidence was lacking within the medical and dental literature.

This guidance was developed in accordance with the MASCC Guidelines Policy [6]. The Group adopted the National Cancer Institute (NCI) definition of advanced cancer (i.e. “cancer that has spread to other places in the body and usually cannot be cured or controlled with treatment”) [24], and data was included from studies involving patients receiving disease-oriented/modifying treatment and especially patients receiving symptom-oriented treatment (i.e. palliative care). Other terms utilised within this review include “end-of-life” (i.e. the last year of life) [25] and “terminal phase” (i.e. the last days to weeks of life) [26].

A search strategy for Medline was developed (Appendix 1), and the Cochrane Database of Systematic Reviews, the Cochrane Oral Health Group’s inventory of systematic reviews and the Cochrane Central Register of Controlled Trials (CENTRAL) were explored for relevant systematic reviews/randomised clinical trials, respectively [27]. The review of the published literature was restricted to papers written in English and to papers relating to adult (> 18 years) humans.

All abstracts identified by the search of Medline were downloaded into a reference management software package (EndNote X9, Clarivate™). These abstracts were independently assessed for relevance by two authors (JJ, AD), and if one author deemed the abstract relevant, then the full text of the article was obtained. The criteria utilised were (a) relevant population; (b) relevant/common clinical problem and (c) relevant/non-specialist treatment intervention. The full text articles were independently assessed for inclusion by the same two authors (JJ, AD). The criteria utilised were (a) relevance to guidance and (b) quality of contents. Moreover, these two authors (JJ, AD) were involved in assessing the systematic reviews in the Cochrane Database of Systematic Reviews/Cochrane Oral Health Group’s inventory of systematic reviews. All of the authors were involved in assessing the randomised controlled trials in CENTRAL.

Generic guidance was often based on standard (evidence-based) dental and oral medicine practice, but specific guidance was invariably restricted to data arising from research studies involving patients with advanced cancer. Guidance was characterised by a level of evidence (i.e. I–V) and a “category of guideline” (i.e. “recommendation”, “suggestion” or “no guideline possible”) (Appendix 2) [6]. All of the authors agree with the final content of the guidance, including the levels of evidence and the categories of guideline.

## Results

The Medline search (original search—24th August 2020; updated searches—29th March 2021 and 5th January 2022) produced 9802 references, and 197 full text articles were retrieved/reviewed. The Cochrane Central Register of Controlled Trials search (search dates same as Medline) produced 6241 references, of which 27 were deemed relevant, including 15 unique studies (c.f. Medline). The analogous searches of the Cochrane Database of Systematic Reviews (search limited to “cancer”) identified one review, which was not identified in the Medline search; the analogous searches of the Cochrane Oral Health Group inventory of systematic reviews (search not limited to cancer) identified 11 potentially relevant reviews, five of which were not identified in the Medline search.

On the basis of the available evidence, the Group generated 12 generic “suggestions”, as well as three problem-specific “recommendations” and 14 problem-specific “suggestions” (see below).

## Generic suggestions

The generic suggestions are summarised in Box 1. The problem-specific recommendations/suggestions are included in the main text. Additionally, the Group recommends that pharmacological interventions are used in accordance with their Summary of Product Characteristics, i.e. that prescribers follow the prescribing guidance (e.g. dose, schedule) and take note of relevant contraindications, cautions, interactions and adverse effects.

Box 1 Generic suggestions about oral care in patients with advanced cancer.

**Table:** 

1—All patients with advanced cancer should be regularly assessed for oral problems (Category of guideline – suggestion; Level of evidence—V) 2—All patients need a regular oral hygiene regimen, and dependent patients need appropriate support with oral hygiene (Category of guideline – suggestion; Level of evidence—V) 3—The management of oral problems should primarily involve treatment of the underlying cause (with appropriate symptom control) (Category of guideline – suggestion; Level of evidence—V) 4—The management of oral problems should be individualised (Category of guideline – suggestion; Level of evidence—V) 5—The management of oral problems should be evidence-based and/or based upon established principles from dentistry/oral medicine (Category of guideline – suggestion; Level of evidence—V) 6—Relevant treatments/interventions should be available in all settings wherever possible (Category of guideline – suggestion; Level of evidence—V) 7—All patients with oral problems should be regularly reassessed (Category of guideline – suggestion; Level of evidence—V) 8—Patients with resistant oral problems should be referred to a specialist for further management (Category of guideline – suggestion; Level of evidence—V) 9—Dental professionals should be members of the extended oncology and palliative care multidisciplinary teams (Category of guideline – suggestion; Level of evidence—V) 10—Oral care in the terminal phase should focus on patient comfort (Category of guideline – suggestion; Level of evidence—V) 11—Oral care in the terminal phase is not a substitute for clinically assisted hydration (Category of guideline – suggestion; Level of evidence—V) 12—Oral care should be an integral component of medical and nursing curricula (undergraduate, postgraduate) (Category of guideline – suggestion; Level of evidence—V)	

### All patients with advanced cancer should be regularly assessed for oral problems (Category of guideline – suggestion; Level of evidence—V)

As discussed, oral problems are practically universal in patients with advanced cancer, and therefore all such patients should be regularly assessed for such problems [28]. The objectives of assessment are to determine (a) the presence of oral problems; (b) the impact of these problems; (c) the cause of these problems; (d) factors that may affect the choice of intervention (i.e. patient-related factors, e.g. co-morbidities; disease-related factors, e.g. prognosis) and (e) patient preference for interventions. Inadequate assessment may result in initiation of inappropriate or contra-indicated interventions.

Assessment primarily involves taking a focussed history and performing a systematic oral examination: in some cases, clinical evaluation may require confirmatory diagnostic investigations (e.g. microbial cultures, dental images). Older generic oral assessment tools are invariably not appropriate/not validated in patients with advanced cancer (e.g. Oral Assessment Guide) [29]. However, an oral symptom assessment tool has been developed/validated in this group of patients (Oral Symptom Assessment Scale) [13], and newer generic tools may also have a role in patients with advanced cancer (e.g. EORTC QLQ-OH15) [30].

Patients should be questioned about common oral symptoms (Table 1), since they may not report such symptoms spontaneously (even when they cause distress) [3]. Importantly, patients should be examined for the presence of oral conditions, even in the absence of oral symptoms. A good direct light source is essential to facilitate adequate visualisation. Gloves must be worn during the examination, and a gloved finger (or a “tongue depressor”/similar implement) used to retract tissues to again facilitate adequate visualisation [31]. Patients with dentures should have these removed during the examination (as they may obscure pathology). Moreover, it is important that denture stability is assessed, and that the denture is examined for any related problems (e.g. poor hygiene, rough edges).

The assessment of oral problems should be responsibility of all healthcare professionals involved in the care of patients with advanced cancer (and not delegated, or assumed to be delegated, to specific groups of healthcare professionals). However, patients with complex oral problems and those relating to the dentition, periodontal tissues and dental prostheses should be referred in a timely manner to relevant dental professionals for specialist treatment.

### All patients need a regular oral hygiene regimen, and dependent patients need appropriate support with oral hygiene (Category of guideline – suggestion; Level of evidence—V)

Good oral hygiene reduces the risk of developing many oral problems in patients with advanced cancer (e.g. halitosis, oral infections). The principles of good oral hygiene are outlined in Box 2 [32]. Care must be taken when assisting patients with their oral hygiene, and gloves and a face mask may be indicated when undertaking oral care (infection control measure). The principles of denture care are outlined in Box 3 [32, 33]. Patients with significant oral hygiene problems, particularly those with “complex” dental restorations, or significant dental/oral conditions, should be referred to relevant dental professionals for specialist treatment.

Box 2 Daily oral hygiene measures [32]

**Table:** 

▪Cleaning of the teeth should be done twice daily using a small headed, soft texture, nylon filament toothbrush and a fluoridated toothpaste. Toothpaste should contain at least 1000 ppm fluoride, but patients with salivary gland hypofunction (especially patients with radiation-induced salivary gland hypofunction) require a toothpaste with 5000 ppm fluoride as well as additional fluoride supplements (e.g., rinses, gels) ▪Alternative toothbrushes/adapted toothbrushes may be needed in some patients (e.g., patients with oral discomfort, patients with disabilities) ▪Toothbrushes should be replaced every 3 months, or when the filaments become misshapen, or when there has been an oral infection ▪Alternative toothpastes/water alone may be needed in some patients (e.g., patients with oral discomfort, patients with dysphagia). A variety of toothpastes are available, including non-foaming (“SLS-free”) options, mint-free options and toothpastes for “sensitive teeth” ▪Chemical plaque control should be utilised in patients unable to clean their teeth (e.g., chlorhexidine mouthwash). Chlorhexidine will prevent the development of plaque, but will not remove plaque in situ (which needs to be removed mechanically). Chlorhexidine is used twice daily ▪Interdental cleaning should be done once daily using dental floss, dental tape or interdental brushes ▪Cleaning of the oral mucosa should be done after every meal and involves either rinsing the mouth with water, or using a moistened gauze (if the patient is unable to rinse their mouth with water)	

Box 3 Daily denture hygiene measures [32, 33]

**Table:** 

▪Dentures should be removed overnight [32, 33] ▪Dentures should be cleaned outside the mouth, and over a bowl/sink of water (to prevent damage if dropped) [32] ▪Cleaning involves once daily (usually at night) mechanical cleaning using a denture brush or a toothbrush, and a non-abrasive denture cleanser (but not toothpaste) [33]. Alternative options include using a nailbrush, and soap and water [32]. Dentures should be rinsed after meals [32] ▪Cleaning also involves once daily (usually overnight) biochemical cleaning using a commercial denture-cleansing solution [33]. Alternative options include using dilute sodium hypochlorite (non-metallic dentures), or chlorhexidine (all dentures) [32]. The latter should be considered in patients with oral infections. Dentures should be rinsed after cleaning [32] ▪It is important to also clean any remaining teeth, periodontium and the oral mucosa (see Box 2) [32, 33]	

### The management of oral problems should primarily involve treatment of the underlying cause (with appropriate symptom control) (Category of guideline – suggestion; Level of evidence—V)

Many oral problems have potentially treatable underlying causes. In such cases, the optimal management is treatment of the underlying cause, although patients may also require symptomatic treatment. If the underlying cause is not treated, it is likely that the oral problem will persist (and even worsen). Moreover, there is an increased risk of developing complications. For example, halitosis may be the result of periodontal disease [34], and non-specific measures to manage halitosis may not resolve this problem (see below). Additionally, uncontrolled periodontal disease may progress to cause important complications such as local infection (abscess), systemic infection (pneumonia), and/or loss of dentition.

### The management of oral problems should be individualised (Category of guideline – suggestion; Level of evidence—V)

As with other problems in this group of patients, the management of oral problems should be individualised. Patients should receive the “optimal” management, which invariably involves treatment of the underlying cause (point 3). However, interventions may need to be amended as a result of the patient’s performance status, their co-morbidities and especially their personal preferences. For example, the optimal management of poorly fitting dentures is to replace the relevant denture. However, relining the denture is an alternative strategy and can be performed at the bedside/in the patient’s home [35].

### The management of oral problems should be evidence-based and/or based upon established principles from dentistry/oral medicine (Category of guideline – suggestion; Level of evidence—V)

Unfortunately, oral care within clinical practice (palliative care) is often based on “anecdotal remedies” [36], with little regard for the established principles of oral care developed within dentistry/oral medicine. Thus, many recommended interventions are relatively ineffective, or completely inappropriate, for their stated purpose(s) (Table 3) [2].

**Table 3:** Inappropriate oral care interventions

Intervention	Indication	Comment
Ice chips/cubes	Xerostomia [37–39]	Ice is frozen water and rapidly melts when placed in the mouth. Ice (like water) provides “temporary and inadequate relief” [40]
Glycerol (+ / − lemon) solution/swabs	Xerostomia [2]	Glycerol has a “drying action” on the oral mucosa [41]. Lemon is acidic (ascorbic, citric acid) and should not be used in patients with salivary gland hypofunction/SGH (especially dentate patients)—acidic products cause oral discomfort and promote dental demineralisation (“erosion”), dental caries and oral candidosis [1]
Acidic artificial salivas	Xerostomia [38]	Acidic products should not be used in patients with SGH (see above)
Pineapple chunks	Dirty mouth [42] Xerostomia [37, 38, 42]	Pineapple is acidic (ascorbic acid), and such products should not be used in patients with SGH
Vitamin C tablets	Dirty mouth/teeth [39, 42] Xerostomia [43]	Vitamin C tablets are acidic (ascorbic acid, often with citric acid), and such products should not be used in patients with SGH
Sweets/candies	Xerostomia	Sweets/candies contain sugars, which cause dental caries (especially in patients with SGH) Sweets/candies may be acidic, and such products should not be used in patients with SGH

### Relevant treatments/interventions should be available in all settings wherever possible (Category of guideline – suggestion; Level of evidence—V)

All patients should have access to the optimal treatment for their oral problems, although treatment needs to be individualised according to the patient’s condition, their care setting and their ability to attend other care settings (e.g. dental surgery, hospital). Nevertheless, most common interventions can be provided in any care settings, and some dental procedures can be performed at the bedside/in the patient’s home (“domiciliary dental care”) [35].

### All patients with oral problems should be regularly reassessed (Category of guideline – suggestion; Level of evidence—V)

All patients with oral problems need to be reassessed in a timely manner (after initiation of treatment), and regularly going forward [28]. The objectives of re-assessment are to determine (a) changes in the clinical condition; (b) adherence with any treatment; (c) the effectiveness of the treatment and (d) the tolerability of the treatment. Inadequate reassessment may result in continuation of ineffective interventions and so persistence or progression of the oral problem.

Patients with recurrent oral problems often have an ongoing underlying cause(s). For example, oral candidosis is often associated with xerostomia/salivary gland hypofunction in patients with advanced cancer [16, 17], and although the clinical infection may respond to a course of antifungal medication, it invariably reoccurs after a short period of time (if the salivary gland hypofunction is not adequately managed—see the “Dry mouth (xerostomia; salivary gland hypofunction)” section).

### Patients with resistant oral problems should be referred to a specialist for further management (Category of guideline – suggestion; Level of evidence—V)

Patients with ongoing oral problems should be referred to an appropriate specialist for further investigation and management (e.g. experienced dental professional, special care dentist, oral medicine professional), especially when the problem is unexplained, is unresponsive (to primary treatment) and/or worsens over time.

### Dental professionals should be members of the extended oncology and palliative care multidisciplinary teams (Category of guideline – suggestion; Level of evidence—V)

Dental professionals are essential members of the oncology multidisciplinary team [44], particularly in terms of preventing and managing the oral complications of anticancer treatment. Similarly, dental professionals are essential members of the extended palliative care multidisciplinary team [45], although there is limited information on their actual roles within these teams [46]. Clearly, an important role is to provide specialist management of complex oral problems and those relating to the dentition, periodontal tissues and dental prostheses [46]. However, an equally important role is to provide training to non-specialist healthcare professionals on maintaining oral hygiene and managing oral problems.

### Oral care in the terminal phase should focus on patient comfort (Category of guideline – suggestion; Level of evidence—V)

Oral care often takes centre stage during the terminal phase (i.e. last days/weeks of life), although the objective outcomes of regular end-of-life “mouth care” regimens are frequently disappointing [47]. The primary aim of oral care should be to maintain oral comfort, rather than to maintain pristine oral hygiene [1]. Indeed, oral hygiene measures often need amending during this period due to patient-related factors. Oral care should be paused and/or discontinued, if there is any indication that it causes distress or discomfort to the patient [1].

Oral care is often delegated to families during the terminal phase. Some family members welcome this task, whilst others find it difficult and/or distressing. It is important, if appropriate, that families are given the opportunity to provide oral care [1]. Equally, it is important that families are not coerced into providing oral care. Furthermore, healthcare professionals must provide adequate instructions and ongoing support and supervision [1]: the former includes the goal of oral care, i.e. to maintain oral comfort (see above re pausing/discontinuing oral care).

### Oral care in the terminal phase is not a substitute for clinically assisted hydration (Category of guideline – suggestion; Level of evidence—V)

Dry mouth is a common problem in patients at the end-of-life, and small amounts of water (“mouth care”) may temporarily relieve this symptom. Thirst is a less common problem in this group of patients, and again small amounts of water may again temporarily relieve this symptom [48]. However, thirst often reflects dehydration, and small amounts of water will not reverse dehydration (or equally maintain hydration). Hence, oral care should be considered a comfort measure, and not a substitute for clinically assisted hydration (in applicable patients).

### Oral care should be an integral component of all medical and nursing curricula (undergraduate and postgraduate) ( Category of guideline—suggestion; Level of evidence - V)

Medical and nursing curricula generally include minimal education on oral care and/or oral problems [49]. However, given the prevalence of oral problems, and their relevance of oral problems to general health, these topics should be an integral component of all medical and nursing curricula worldwide. Moreover, dental professionals should be involved in curricula development, and particularly the development of educational resources (to ensure that the content is appropriate).

The evidence base for the above generic suggestions is limited, and the evidence base for the following specific suggestions is equally limited. Hence, further research is required in this group of patients. Research in patients with advanced cancer is challenging but not impossible (as researchers have consistently demonstrated) [50–53]. In the meantime, oral care in patients with advanced cancer can be based on evidence derived from other groups of patients (and not on clinical anecdotes) [36].

## Specific recommendations

### Dry mouth (xerostomia; salivary gland hypofunction)

#### Terminology

Xerostomia refers to a subjective sensation of dryness of the mouth, whilst salivary gland hypofunction refers to an objective decrease in salivary gland secretion(s).

#### Aetiology

There are many potential causes for a dry mouth in patients with advanced cancer [1]. However, the main cause relates to the adverse effects of medication used within general medicine and oncology/palliative care [54].

#### Management

Management should include treatment of the underlying cause (if possible), e.g. discontinuation of offending medication *(Category of guideline—suggestion: Level of evidence—V)*. Symptomatic management involves both non-pharmacological and pharmacological interventions [55].

Non-pharmacological interventions include water, artificial salivas *(Category of guideline—recommendation: Level of evidence—II, i.e. mucin-based artificial saliva)* [50–52], chewing gum *(Category of guideline—recommendation: Level of evidence—II)* [52] and acupuncture *(No guideline possible)* [56, 57]. Artificial salivas tend to have a longer duration of action than water [1], and a number of commercial products are available, which differ in formulation (e.g. spray, gel), pH (e.g. acidic, neutral), lubricant (e.g. mucin, carboxymethylcellulose) and additional ingredients (e.g. flavourings, fluoride). Ideally, an artificial saliva should have a neutral pH (to limit demineralisation of the teeth) and contain fluoride (to enhance remineralisation of the teeth).

Pharmacological interventions include pilocarpine (*Category of guideline—recommendation: Level of evidence—II)* [52] and related products (e.g. bethanechol, cevimeline). (Currently, there is no evidence to support the use of intraoral pilocarpine eyedrops in this condition [58]).

Saliva stimulants (e.g. sugar-free chewing gum, pilocarpine) are generally preferred to saliva substitutes (e.g. water, artificial salivas) when the salivary glands can be stimulated, since they are more effective in relieving xerostomia, and they should improve the other problems associated with salivary gland hypofunction (see above) [1]. It should be noted that the evidence on managing xerostomia/salivary gland hypofunction per se is of limited quality [59, 60].

### Taste disturbance

#### Terminology

Patients with advanced cancer report a variety of taste disturbances, which include ageusia (an absence of taste sensation), hypogeusia (a decrease in taste sensation), hypergeusia (an increase in taste sensation) and dysgeusia (a distortion of normal taste sensation) [61, 62].

#### Aetiology

There are several potential causes of new-onset taste disturbance in patients with advanced cancer, including salivary gland hypofunction, poor oral hygiene, medication [63] and nutritional deficiencies (e.g. zinc). Moreover, taste disturbance may be an ongoing complication of the cancer and/or the oncological treatment (e.g. systemic chemotherapy, head and neck radiotherapy) [64].

#### Management

Management should include treatment of the underlying cause (if possible), e.g. treatment of salivary gland hypofunction *(Category of guideline—suggestion: Level of evidence—IV)* [65]. Symptomatic management primarily involves non-pharmacological interventions.

Patients with taste disturbance should be reviewed by a dietitian, and a personalised nutritional plan developed *(Category of guideline—suggestion: Level of evidence—V)*. Strategies that may be useful include utilisation of foods that taste “good”; avoidance of foods that taste “bad”; enhancing the taste of the food using salt, sugar and other flavourings; moistening the food and addressing the presentation, smell, consistency and temperature of the food, i.e. paying attention to other aspects of flavour *(Category of guideline—suggestion: Level of evidence—V)* [66, 67].

Pharmacological interventions reported to be effective in different cohorts of patients with cancer, include zinc supplements *(no guideline possible)* [68], dronabinol *(Category of guideline—suggestion: Level of evidence—II, i.e. pilot study)* [69], megestrol acetate *(no guideline possible)* [70] and clonazepam *(no guideline possible)* [65].

### Oral discomfort/pain

#### Aetiology

There are several potential causes of new onset oral discomfort/pain in patients with advanced cancer, including salivary gland hypofunction [54], mucosal conditions (i.e. infection/inflammation/ulceration) [12, 71], dental-related problems, denture-related problems [71], neurological conditions and pain referred from adjacent structures.

#### Management

Management should include treatment of the underlying cause (if possible), e.g. relining, adjusting, or replacing poorly fitting dentures *(Category of guideline—suggestion: Level of evidence—V)*. Symptomatic management primarily involves pharmacological interventions, which may include both topical agents and systemic analgesics. The optimal analgesic regimen depends on the aetiology of the pain (cancer-related, coexisting condition), the pathophysiology of the pain (nociceptive, neuropathic) and a variety of patient-related factors (e.g. co-morbidities, personal preference) *(Category of guideline—suggestion: Level of evidence—V)* [72].

Bland rinses (e.g. normal saline) can often provide some relief for patients with oral ulceration, including patients with oral mucositis. Similarly, “coating” agents can provide some relief for patients with oral ulceration, particularly in patients with pain on eating/drinking (“contact”/incident pain). However, many patients with oral ulceration have associated inflammation, and so merely covering the ulcer(s) does not relieve the discomfort. Topical analgesics can be highly effective in some cases, and options include benzydamine hydrochloride solution/spray (anti-inflammatory), oral morphine solution (opioid) and doxepin solution (antidepressant) [73]. Topical analgesics are less likely to cause systemic adverse effects, and so are often considered as first line treatments. Local anaesthetics have a limited role, and care must be taken to prevent inadvertent aspiration. Equally, topical steroids have a limited role (e.g. recurrent aphthous stomatitis), and care must be taken to exclude oral problems that might worsen with steroids (e.g. oral infections).

Systemic analgesics can also be effective, but there are limited reasons for chronic administration (due to concerns about adverse effects).

Interventional techniques are another option in highly select cohorts of patients (e.g. patients with intraoral tumours).

### Halitosis

#### Terminology

Patients with genuine halitosis (i.e. objective evidence of malodour) can be subclassified into those with physiological halitosis (i.e. no evidence of underlying condition) and pathological halitosis (i.e. evidence of underlying condition).

#### Aetiology

Physiological halitosis is the most common type: the cause of physiological halitosis is the bacterial putrefaction of food debris, epithelial cells, blood cells and saliva within the oral cavity (and particularly on the posterior part of the dorsum of the tongue) and poor/limited oral hygiene. The cause of pathological halitosis may be an infection, inflammation or malignancy. In ~ 90% such cases, the source of the malodour is the oral cavity, but other potential sources include the upper respiratory tract (nose, sinuses), the lower respiratory tract, the gastrointestinal tract or a systemic metabolic problem.

#### Management

Management depends on the type of halitosis: in patients with physiological halitosis, it primarily involves oral hygiene measures, whilst in patients with pathological halitosis, it primarily involves treatment of the underlying cause (if possible), e.g. antibiotics for infections *(Category of guideline—suggestion: Level of evidence—V)*.

The management of physiological halitosis includes [74] *(Category of guideline—suggestion: Level of evidence—V)* (a) generic oral hygiene measures (see above); (b) avoidance of odorous foodstuffs (e.g. garlic, onions); (c) alcohol cessation; (d) smoking cessation; (e) measures to reduce bacterial numbers (e.g. tongue cleaning, chlorhexidine—mouthwash); (f) measures to reduce bacterial substrate (e.g. tongue cleaning, “professional” dental/periodontal cleaning) and (g) measures to convert offensive volatile sulphur compounds to inoffensive non-volatile compounds (e.g. zinc salts—toothpaste, mouthwash; sodium bicarbonate/baking soda – toothpaste). Many oral care products are available to manage halitosis, and those with specific properties (see above) should be prescribed in preference to those with simply “masking”/cosmetic properties *(Category of guideline—suggestion: Level of evidence—V)*. It should be noted that the evidence on managing halitosis in general is of limited quality [75].

### Oral candidosis

#### Aetiology

*Candida* species are opportunistic microorganisms, and so oral candidosis usually occurs as a result of changes in host systemic and/or intraoral factors [76]. In patients with advanced cancer, oral candidosis has been found to be specifically associated with poor performance status, salivary gland hypofunction, presence of denture and use of systemic steroids [16, 17]. However, other factors may also have a role in the development of oral candidosis in this group of patients (e.g. immunosuppression, systemic antibiotics, mucosal damage) [77].

#### Management

The management of confirmed oral candidosis primarily involves the use of antifungal medication (i.e. polyenes, azoles). However, antifungal medication (especially azoles) should preferably be reserved for patients with laboratory-confirmed oral candidosis *(Category of guideline—suggestion: Level of evidence—V)*, since other oral conditions can have similar features, and antifungal resistance is reported to be common in this group of patients [78–80]. Importantly, antifungal medication should be combined with treatment of predisposing factors (see above) *(Category of guideline—suggestion: Level of evidence—V)*, since many patients will recur after the course of antifungal medication is completed (due to persistence of predisposing factors).

A variety of topical and systemic antifungal medications are available for treating oral candidosis. The choice of treatment depends on a number of factors [1] *(Category of guideline—suggestion: Level of evidence—V)*: (a) extent of disease – topical agents are appropriate for treating localised disease, whilst systemic agents are more appropriate for treating multifocal/generalised disease; (b) immunocompetence – systemic agents are more appropriate for treating immunosuppressed patients; (c) drug resistance – resistance to the polyenes is uncommon, although resistance to the azoles is relatively common; (d) concomitant disease – azoles have a number of relative/absolute contraindications; (e) concomitant medication – systemic azoles have a number of drug interactions; (f) patient preference; (g) ease of use – topical agents are more difficult to use, and efficacy is dependent on correct usage (i.e. contact with lesions) and (g) patient adherence. (Currently, there is no robust evidence to support the use of single doses of fluconazole in this condition [81] *(no guideline possible)*).

Other options for patients with recurrent/resistant oral candidosis, or patients with difficulties in using conventional antifungal medication, include chlorhexidine *(no guideline possible)* [82] and tea tree oil *(no guideline possible)* [83].

It should be noted that the successful management of denture-related stomatitis (with/without angular cheilitis) depends on a combination of antifungal drug treatment, and disinfection of the denture (see Box 3) *(Category of guideline—suggestion: Level of evidence—V)*. When the patient wears a denture, the denture should be treated as well to prevent reinfection of the oral cavity, e.g. by immersing it in a chlorhexidine mouth rinse.

## Conclusion

This guidance provides an evidence-based framework for the management of common oral problems in patients with advanced cancer, although every patient requires individualised management (based upon a thorough assessment). Many of the recommendations are based on low levels of evidence, and/or evidence extrapolated from other groups of patients with cancer. However, patients with advanced cancer are in many ways a unique group, and so further research is warranted in this specific group in order to improve their oral care (and quality of life).

## Data Availability

Not applicable.

## Appendix 1 MEDLINE search strategy

1. Oral care—keyword

**or (search terms 2–56)**

2. Oral medicine—MESH
3. Dental care—MESH
4. Oral health—MESH
5. Oral assessment—keyword
6. Oral hygiene—MESH
7. Toothbrushing—MESH
8. Interdental cleaning—keyword
9. Mouthwashes—MESH
10. Mouth—MESH
11. Mouth diseases—MESH
12. Lip diseases—MESH
13. Cheilitis—MESH
14. Tongue diseases—MESH
15. Mouth mucosa—MESH
16. Oral mucosa—keyword
17. Stomatitis—MESH
18. Oral ulcer—MESH
19. Tooth—MESH
20. Tooth diseases—MESH
21. Toothache—MESH
22. Dentin sensitivity—MESH
23. Tooth erosion—MESH
24. Dental plaque—MESH
25. Dentures—MESH
26. Dental prosthesis—MESH
27. Denture retention—MESH
28. Stomatitis, denture—MESH
29. Dental devices, home care—MESH
30. Jaw diseases—MESH
31. Mastication—MESH
32. Trismus—MESH
33. Salivary gland diseases—MESH
34. Xerostomia—MESH
35. Dry mouth—keyword
36. Taste disorders—MESH
37. Taste disturbance—keyword
38. Ageusia—MESH
39. Dysgeusia—MESH
40. Hypogeusia—keyword
41. Facial pain—MESH
42. Jaw pain—keyword
43. Oral pain—keyword
44. Mouth pain—keyword
45. Glossalgia—MESH
46. Burning mouth syndrome—MESH
47. Oral infections—keyword
48. Oral bacterial infections—keyword
49. Dental caries—MESH
50. Periodontal diseases—MESH
51. Gingivitis—MESH
52. Oral fungal infections—keyword
53. Candidiasis, oral—MESH
54. Oral candidosis—keyword
55. Oral viral infections—keyword
56. Halitosis—MESH

**and (search terms 57–58)**

57. Neoplasm—MESH

**or**

58. Cancer—keyword

## Appendix 2 MASCC criteria for grading recommendations [6]

Levels of evidence.

**Table:**

I	Evidence obtained from meta-analysis of multiple, well-designed, controlled studies; randomised trials with low false-positive and false-negative errors (high power)
II	Evidence obtained from at least one well-designed experimental study; randomised trials with high false-positive and/or false-negative errors (low power)
III	Evidence obtained from well-designed, quasi-experimental studies, such as nonrandomized, controlled single-group, pretest–posttest comparison, cohort, time or matched case–control series
IV	Evidence obtained from well-designed, non-experimental studies, such as comparative and correlational descriptive and case studies
V	Evidence obtained from case reports and clinical examples

Categories of guidelines.

**Table:**

Recommendation	Reserved for guidelines that are based on Level I or Level II evidence
Suggestion	Used for guidelines that are based on Level III, Level IV and Level V evidence; this implies panel consensus on the interpretation of this evidence
No guideline possible	Used when there is insufficient evidence on which to base a guideline; this implies (1) that there is little or no evidence regarding the practice in question, or (2) that the panel lacks consensus on the interpretation of existing evidence
